# The PortaLyzer, a DIY tool that allows environmental DNA extraction in the field

**DOI:** 10.1016/j.ohx.2022.e00373

**Published:** 2022-10-27

**Authors:** Charlie Peck, Faith Jackobs, Emmett Smith

**Affiliations:** Computer Science - Earlham College, 801 National Road West, Richmond, IN 47374, United States; Quantitative and Computational Biosciences Program, Baylor College of Medicine, 1 Baylor Plaza, Houston, TX 77030, United States; Biology - Earlham College, 801 National Road West, Richmond, IN 47374, United States

**Keywords:** Environmental DNA, Field DNA extraction, Bead-beating, Portable, eDNA, environmental DNA, °C, degrees Celsius, USD, United States Dollars, OPM, Oscillations per minute

## Abstract

The PortaLyzer is a portable homemade device that allows researchers to perform bead-beating steps commonly found in environmental DNA (eDNA) extraction protocols in the field without access to power. This allows researchers to preserve *in situ* organism abundance by beginning eDNA extraction quickly. The PortaLyzer is composed of a variable speed, battery-powered multi-tool and a vortexer adapter plate. We used the PortaLyzer, in conjunction with the Qiagen DNEasy PowerSoil Pro kit, to successfully field process samples taken from the forelands of the Sólheimajökull and Kvíárjökull glaciers in Iceland, as well as soil samples acquired from a prairie in Indiana. Additionally, we provide evidence that samples held in Buffer CD2 of the DNEasy PowerSoil Pro Kit may be transported to traditional lab spaces and processed up to one month after the initial protocol steps, and still provide an equivalent DNA quality and abundance yield as those processed the same day. These improvements to DNA extraction protocols give researchers more flexibility while sampling, shipping and processing eDNA samples.

## Highlights

• The homemade PortaLyzer can replace the vortex for environmental DNA extractions.
• The PortaLyzer is easy to assemble, portable, and runs on battery power.
• Storing samples at 4 °C in Buffer CD2 for one month doesn’t affect DNA quality.
• Begin environmental DNA extraction entirely in the field.

## Graphical abstract

**Figure:**
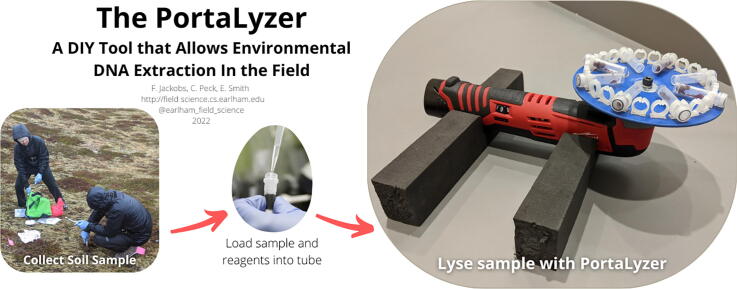

## Specifications table

**Table:** 

Hardware name	PortaLyzer
Subject area	Biological sciences (e.g., microbiology and biochemistry)
Hardware type	Biological sample handling and preparation
Closest commercial analog	FastPrep-24 (approximately $9,000USD), TerraLyzer (no longer for sale), Qiagen PowerLyzer 24 Homogenizer ($4,500–6,000), the Qiagen TissueLyzer II ($4,000–6,000 USD), Benchmark Scientific BeadBlaster Microtube Homogenizer ($8,295 USD), Vortex-Genie + Adapter ($500 USD)
Open source license	Creative Commons Attribution 4.0 International (CC BY 4.0)
Cost of hardware	$200 USD
Source file repository	Not applicable.

## Hardware in context

Environmental DNA (eDNA) is any DNA that can be found in the environment, including soil, water, on rocks or plant leaves or even floating in the air. Detection and analysis of eDNA is rapidly becoming commonplace since it allows researchers to non-invasively monitor biodiversity, a critical ability in the face of our warming climate (reviewed in [1], [2]). Soil microbiomes are of particular interest for many reasons, including their importance to plant succession and their responses to climate change [3], [4], [5]. We work on extracting environmental DNA (eDNA) from soils in Iceland to study the effects of climate change on the soil microbiome. Though our sampling locations in Iceland are not extremely remote, we have focused on creating a robust, effective eDNA extraction protocol that can be started in the field. To that end, we have tested a new device we are calling the PortaLyzer, described in this paper, and tested whether storing DNA for up to one month at 4 °C before extraction is complete affects the DNA quality [6]. The PortaLyzer is a combination multi-tool and vortexer adapter that can perform the bead-beating step of eDNA extraction protocols using battery power only. Using this device, in combination with hand- or battery-powered centrifuges, frees researchers from the bench, allowing them to perform the first part of eDNA extraction in the field. The total cost of materials to build your own PortaLyzer is just under $200 USD. The device is easy to create and use. Often it is easier to transport extracted DNA across international borders than soil samples. We hope the PortaLyzer will enable more scientists to engage in the exciting field of environmental DNA and support extended and higher quality field sampling.

Free eDNA in soil can be tightly bound to soil particles, while intracellular DNA from organisms is contained within a durable cell membrane, and often an additional cell wall [7]. Chemical and physical disruption is needed to break open cells and detach DNA from soil particles, and many eDNA extraction methods include a vigorous bead-beating step [8], [9]. Some soil types retain eDNA better than others. The most prevalent Icelandic soil type is the Andosol, which is rare in northern Europe and other circumpolar regions but common to volcanic landscapes [7], [10]. Extracting microbial DNA from Andosols can be difficult due to DNA re-binding to soil particles (adsorption) and the presence of “macroaggregates” of bacteria inside soil pores, exacerbated by Andosol minerals’ large surface area [7], [8], [10]. One way to increase DNA yield from Andosols is to add skim milk, which reduces DNA re-binding to clay particles [11]. In order to successfully separate and extract eDNA from cells and soil particles, a bead-beating step is recommended [9]. In a bead-beating step, small amounts of soil is added to a tube containing multiple small beads and a buffer and vigorously shaken for 10–20 minutes to lyse cell walls and membranes as well as detach eDNA from soil particles. This is referred to as “homogenization.” To enable homogenization, machines such as a vortex with a specific adapter are commonly used (see Qiagen Vortex Adapter for 24 (1.5–2.0 ml) tubes (cat. no. 13000-V1-24)). Adapters are generally available for 1.7 mL and 5 mL tubes, though it is possible to 3D print an adapter that fits a different tube size (see https://3dprint.nih.gov/discover/3dpx-014901 for an example of an open-source adapter).

Bacterial populations can shift quickly in response to changes in their environment. This is a concern for extracting bacterial DNA from soils because as soon as the soil is removed from the ground bacterial populations may begin to change, resulting in data that does not match authentic bacterial population abundances [12]. To ameliorate this issue, many researchers place samples on dry ice and immediately transport samples to the lab for DNA extraction, or freeze samples at −80℃ until extraction can take place. Other groups have shown that sample preservation in a specific buffer preserves microbial community structure [13]. When working in remote field locations it’s not always possible to acquire and preserve dry ice, nor is lab space and electricity easily accessible. In these instances, starting a DNA extraction protocol in the field is ideal, and some groups create battery- or solar-powered “remote labs” at their field sites [14], [15]. This requires transporting extra, sometimes heavy and bulky, equipment. Some commercially available homogenization devices are small enough to be portable, but all (that we know of) require electricity. The most basic device used to perform the important bead-beating step is a vortexer (such as the Vortex-Genie) with an adapter for holding tubes horizontally. Many vortexers are small, somewhat portable (though heavy) and affordable ($100–400 USD). Beyond vortexers, there are large machines designed specifically for homogenization. The FastPrep-24™ is a commonly used device that agitates biological samples to homogenize, grind or lyse them. Unfortunately, the FastPrep-24™ costs over $9,000 USD and is definitely not portable. Other commercially available homogenizers have the same price and portability barrier: the Qiagen PowerLyzer 24 Homogenizer ($4,500–6,000), the Qiagen TissueLyzer II ($4,000–6,000 USD), and the Benchmark Scientific BeadBlaster Microtube Homogenizer ($8,295 USD, benchmark.com). To give researchers a field-friendly bead-beating device, Zymo Research developed the TerraLyzer, which cost $1,200 USD, but has discontinued the product. Since then, there have been no other models of a field-based vortexer device, though interest in eDNA field extractions is increasing. Though we do not demonstrate the use of field centrifuges in this paper, readers should be aware of their existence and consider incorporating them into a field workflow (examples include [16], [17] OpenFuge (https://www.instructables.com/OpenFuge/) and the Open Hardware centrifuge project (https://fosh-following-demand.github.io/Open-source-Centrifuge-for-WetLab/)).

The DNEasy PowerSoil Pro Kit sold by Qiagen is recommended for high-quality eDNA extraction from soils and is commonly used among eDNA researchers [18]. The protocol begins with homogenizing the sample. In this step, a small amount of soil is added to PowerBead Pro tubes plus CD1 buffer (supplied in the kit) followed by horizontal vortexing for 10-20 minutes. The sample is then centrifuged to separate debris from the eDNA, the supernatant is transferred to a new tube and Buffer CD2 (supplied in the kit) is added. In a traditional eDNA extraction protocol, these steps must be done in the lab due to the necessity of electricity to run a vortexer and centrifuge. Field models for centrifuges have already been presented [16], [17] (see OpenFuge (https://www.instructables.com/OpenFuge/) and the Open Hardware centrifuge project (https://fosh-following-demand.github.io/Open-source-Centrifuge-for-WetLab/)). The PortaLyzer allows researchers to perform the initial bead-beating step in the field, without needing to store samples on dry or wet ice or transport them to a location with electricity. Additionally, as we show, storing samples in Buffer CD2 for up to one month at 4℃ does not decrease DNA yield or quality. The PortaLyzer and open-source centrifuges provide immense flexibility to anyone working with soil eDNA. Our work demonstrates that researchers can perform the bead-beating and initial centrifugation steps in the field and place samples in buffer CD2 for long-term storage in the fridge, ready for transport.

## Hardware description

**Fig. 1:**
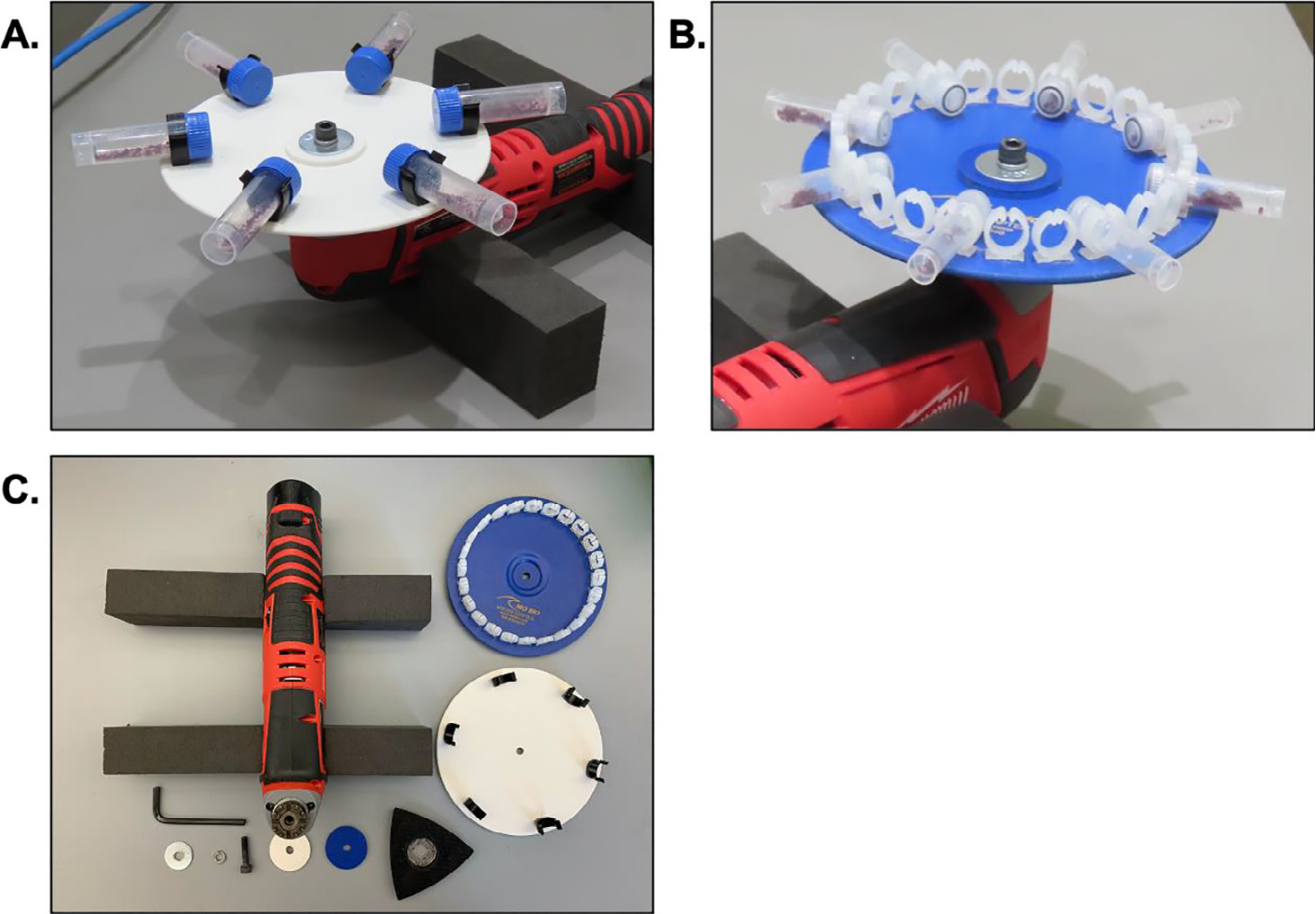
**The PortaLyzer.** (A) With 6 × 5 mL tube vortex plate. (B) With 24 × 2 mL tube vortex plate. (C) All the parts needed for assembly of the PortaLyzer. Note the foam cradles which support the device while in use double as packing material for transportation.

The basis of the PortaLyzer is a battery powered multi-tool to which we affix a standard vortexer plate and a simple bench cradle. The multi-tool's oscillating motion closely mimics that provided by a bench-top vortexer. The frequency is adjustable between 5,000-20,000 oscillations per minute in 2,000 OPM increments. The oscillation angle, or amplitude, is about 3 degrees. This compares to the 600-3200 full eccentric rotations per minute provided by a bench-top vortexer, leading to a similar action within the sample tubes for both devices. In general, the adjustable motion that modern multi-tools provide can be easily converted to meet a variety of sample preparation needs. As illustrated below, vortexer plate adapters which hold varying sample tube sizes can be used depending on the requirements of the DNA extraction protocol **(**Figure 1**)**.

For eDNA extraction, homogenization using chemicals and beads improves yield [8]. This is achieved by random collisions of beads with soil particles and microbial cells, causing cells to burst open and release their DNA into the buffer, which decreases adsorption onto soil particles. The “vortex” action of a vortexer machine is not needed to achieve this, rather, mechanical agitation leads to cell bursting. This agitation is achieved by the multi-tool we employ as the basis of the PortaLyzer.

One battery (supplied with the multitool) powers the PortaLyzer for over one hour of run-time, broken down into three approximately 20 minute intervals at 5,000 OPM. The multitool base is sturdy and does not need special care. The vortexer adapter plate is also sturdy, though care should be taken not to over-tighten the bolt or the plastic plate may warp. The plastic holders that enclose the tubes could be snapped off with hard use, but replacements can be purchased or 3D printed and easily re-installed. If using a 3D printed adapter plate, we suggest taking extra care while using the device, as the type of plastic used for printing may affect the fragility of the adapter plate.

• Improved environmental DNA (eDNA) Extraction
• Field-based eDNA Extraction
• Easier Transport of Samples Across Country Borders

## Design files summary

A three minute video that shows how to assemble the unit from the parts, a one minute video that demonstrates its use in the lab, and a two minute video that demonstrates its use in the field.

**Table:** 

**Design file name**	**File type**	**Open source license**	**Location of the file**
PortaLyzer Assembly Video	Video	Creative Commons Attribution 4.0 International (CC BY 4.0)	https://youtu.be/BqmNYpFdPPY
PortaLyzer Usage Video	Video	Creative Commons Attribution 4.0 International (CC BY 4.0)	https://youtu.be/a0eiRGB9P_o
PoraLyzer Field Usage Video	Video	Creative Commons Attribution 4.0 International (CC BY 4.0)	https://youtu.be/__26bqYdXGU

## Bill of materials summary

**Table:** 

**Designator**	**Component**	**Number**	**Cost per unit -currency**	**Total cost -currency**	**Source of materials**	**Material type**
Motor	a	1	$65 USD	$65 USD	Many sources	Composite
Plate	b	1	$75 USD	$75 USD	Many sources	Plastic
Battery	c	1	$30 USD	$30 USD	Many sources	Composite
Charger	d	1	$20 USD	$20 USD	Many sources	Composite
Bolt	e	1	$0.5 USD	$0.5 USD	Many sources	Steel
Flat washer	f	1	$0.2 USD	$0.2 USD	Many sources	Steel
Split washer	g	1	$0.2 USD	$0.2 USD	Many sources	Steel
Foam	h	1	$5 USD	$5 USD	Many sources	Foam

a) We used a Milwaukee 2426-20 12 Volt Lithium Ion 20,000 OPM Variable Speed Cordless Multi Tool. Similar units will also work, e.g. the Skil PWRCORE 20 oscillating tool and the Kimo L23Q3.
b) Vortexer adapter plate(s) appropriate for the tube size(s) you use. An open source, 3D printable plate can be found at https://3dprint.nih.gov/discover/3dpx-014901
c) Milwaukee 48-59-2401 12 Volt Lithium Ion Battery Charger
d) Powerextra 12V 3000mAh Lithium-ion Replacement Battery (2 pack)
e) 4 mm × 3.75 mm cap bolt, Allen head
f) ¼“ x 1” flat washer
g) ¼“ split lock washer
h) Foam blocks

## Build instructions

Reference the assembly video: https://youtu.be/BqmNYpFdPPY

1) Collect the materials (components a-h, see Figure 1**C**).
2) Collect the tools: Allen wrench (3/16“ or 3 mm), small electric drill, and ¼” or 4 mm drill bit.
3) Using the drill and bit, enlarge the center hole of the plate. Similarly enlarge the center hole of the plastic washer that came with the plate kit.
4) Locate the triangular backing plate that came with the motor kit. In order, place the backing plate (logo side facing the motor), plate (b), plastic washer from the plate kit, ¼“x1” flat washer (f), ¼“ split washer (g); securing them with the Allen bolt (e). Hand-tighten the bolt using the Allen wrench.
5) Cut the foam into support blocks as shown, these act both as a stand when the PortaLyzer is in use and as packing material for transportation.

## Operation instructions

Reference the lab usage video: https://youtu.be/a0eiRGB9P_o

Reference the field usage video: https://youtu.be/__26bqYdXGU

1) Place a charged battery into the motor.
2) If you removed the vortexer plate for packaging/transport, reassemble the unit as described in step 4 of the “Build instructions”.
3) Place the motor into the foam support blocks.
4) Load your sample tubes onto the plate.
5) Adjust the speed using the dial mounted on the side of the motor. Our experience is that setting 2 (5,000 OPM) works well.
6) Turn on the motor, and set your timer for the appropriate lyzing interval based on your protocol.

For safety, ensure that the device is on a level, stable surface while in-use. The PortaLyzer may be stabilized with pre-cut foam blocks (as illustrated in Figure 1) or other materials at hand. Check to make sure the device will not fall off an edge while in operation. One may desire to wear earplugs while the device is in use if working close by. There is no need for eye protection while operating the device.

## Validation and characterization

In June 2021, we tested the PortaLyzer in the field with the DNEasy PowerSoil Pro kit from Qiagen (cat. no. 47014). We took 9 soil samples from various locations in the forefield of the Icelandic glacier Kvíárjökull (KV) and 10 soil samples from various locations in the forefield of the Icelandic glacier Sólheimajökull (Solo). Soil samples were stored at 4℃ for 5-7 days before using the PortaLyzer. We prepared duplicates for each sample, using 0.25 g of soil and following the instructions of the PowerSoil Pro kit until the vortexing step, with the addition of 10 mg of powdered skim milk at the bead beating stage to enhance DNA dissociation from soil [11]. All samples were homogenized using the PortaLyzer for 20 minutes on speed level 2 in place of the 10-20 minute vortexer step in the PowerSoil protocol. After homogenization, half of the samples were fully extracted and purified while in Iceland, on June 14, 2021. The other half of the samples were kept in buffer CD2 for one month at 4 °C. There are few studies on the effect of long-term storage on microbial community composition in soil samples, but others have shown that cold storage (4 °C) up to a month does not significantly impact results [6], [19], [20]. These samples were transported back to the US in a styrofoam box kept cool with frozen vegetables. We finished the DNA isolation using the PowerSoil Pro kit in a college laboratory setting, on July 16, 2021. All samples were run through the DNA Clean + Concentrator Kit from Zymo (cat. no. D4013) before being sent for 16S amplicon library preparation and sequencing.

The PortaLyzer, in conjunction with Qiagen’s DNEasy PowerSoil Pro Kit and Zymo’s DNA Clean and Concentrator Kit, produced quality yield from DNA extraction protocols (expected yields range from 10-200 ng/ul) (Table 1). We measured DNA concentration using the Broad Range dsDNA Assay on the Qubit 3.0. Our average yield for the samples fully processed in Iceland was 70 +/-12 ng/ul. Our average yield for the samples partially processed in Iceland, shipped home and finished at Earlham College was 63 +/-13 ng/ul. The purity of the samples was analyzed using a Nanodrop. The A260/280 is a measure of DNA purity, with a ratio of ∼ 1.8 generally accepted as pure for DNA [21]. The A260/230 ratio indicates the presence of unwanted organic compounds, where values between 2.0-2.2 indicate “pure” nucleic acid [21]. Based on these ratios, our samples were pure but may have minor levels of organic contaminants, even when using the vortexer (Table 1). Regardless, the samples were pure enough for sequencing. We sent extracted DNA to the Center for Genomics and Bioinformatics at Indiana University for 16S V4 amplicon library preparation and sequencing on the Illumina MiSeq with the 600-cycle V3 kit, where we obtained over 10 million reads for each extraction method. (Primers used for sequencing were custom 16SV4 For CGB Primer 5’-CACTCTTTCCCTACACGACGCTCTTCCGATCTTATGGTAATTGTGTGCCAGCMGCCGCGGTA*A-3’ and Custom 16SV4 Rev CGB Primer 5’-GTTCAGACGTGTGCTCTTCCGATCTAGTCAGTCAGCCGGACTACHVGGGTWTCTAA*T-3’ where * indicates a phosphothioate bond). Raw sequencing files for this data can be found at https://www.ncbi.nlm.nih.gov/bioproject/PRJNA883703.

**Table 1:** **DNA Extraction Quality Comparison**. StErr = Standard Error.

**Soil Type: DNA Extraction Method: Location (Year)**	**Average DNA Yield (ng/ul)**	**A260/280 (average)**	**A260/230 (average)**
Brown Andisol: DNEasy PowerSoil Pro Kit with PortaLyzer, plus DNA Clean and Concentrator: full extraction in Iceland (2021)	Average: 70 StErr: 12	Average: 1.92 StErr: 0.2	Average: 1.87 StErr: 0.1
Brown Andisol: DNEasy PowerSoil Pro Kit with PortaLyzer, plus DNA Clean and Concentrator: partial extraction in Iceland (2021)	Average: 63 StErr: 13	Average: 1.82 StErr: 0.32	Average: 2.05 StErr: 0.09
Brown Andisol: DNEasy PowerSoil Pro Kit with vortexer, plus DNA Clean and Concentrator: full extraction in USA (2019)	Average: 33 StErr: 9	Average: 1.84 StErr: 0.02	Average: 0.92 StErr: 0.14
Brown Silt Loam: DNEasy PowerSoil Pro Kit with PortaLyzer: full extraction in USA (2022)	Average: 313 StErr: 33	Average: 1.91 StErr: 0.00	Average: 1.48 StErr: 0.16
Brown Silt Loam: DNEasy PowerSoil Pro Kit with vortexer: full extraction in USA (2022)	Average: 336 StErr: 25	Average: 1.91 StErr: 0.00	Average: 1.67 StErr: 0.15

In 2019, we extracted eDNA from 14 soil samples taken from East Iceland Andisol soils. Soil samples were frozen and shipped to the USA for extraction at Earlham College. We used the Qiagen DNEasy PowerSoil Pro Kit as in 2021, with a vortexer at the bead-beating step. Samples were homogenized for 20 minutes on a vortexer using the Qiagen Vortex Adapter for 24 (1.5–2.0 ml) tubes (cat. no. 13000-V1-24) (the same adapter used with the PortaLyzer). These samples were sent for 16S sequencing at the Center for Genomics and Bioinformatics at Indiana University for 16S V4 amplicon library preparation and sequencing on the Illumina MiSeq with the 600-cycle V3 kit, where we obtained over 1 million reads in total. We present this data to contrast with the 2021 eDNA extractions.

In the summer of 2022 we performed a direct comparison of DNA extraction using the PortaLyzer versus a standard lab vortexer, following the procedures of the DNEasy PowerSoil Pro Kit with no modifications. The vortexer was fitted with the same adapter the PortaLyzer uses (Qiagen Vortex Adapter for 24 (1.5–2.0 ml) tubes). For this comparison, 11 soil samples were collected from a recently planted prairie strip along the north side of Earlham College’s Miller Farm. The soil at this location is a mixture of the Miami-Crosby-Strawn soil types [22], [23], [24]. This soil is a brown silt loam, laid down in glacial moraines at the end of the last Ice Age. It is a rich soil with a moderately acid to neutral pH. The granular structure can vary from moderate medium to moderate fine to weak fine and medium. Both sets of extractions were shaken for 10 minutes on speed 2 with the PortaLyzer and on max speed on the vortexer. We achieved very high yield, with averages of 313 +/-33 and 336 +/-25 ng/ul for the PortaLyzer and vortexer, respectively (Table 1). The DNA had an average A260/280 measurement of 1.91 and an average A260/230 reading below 2.2 for both devices (Table 1). As of this writing, these samples have not been sent for sequencing but based on the high yield and purity of samples we anticipate both methods would yield high quality reads.

We present this data for a comparison between the vortexer and PortaLyzer methods (Table 1, Table 2). We note that the increased number of reads in 2021 may be due to an update the sequencing core made to their library prep procedure. Unfortunately, due to the cost associated with purchasing commercial lysis equipment such as the the FastPrep-24™, PowerLyzer 24 Homogenizer, TissueLyzer II and the BeadBlaster Microtube Homogenizer, we were unable to directly compare the vortexer or PortaLyzer with such devices. However, using the PortaLyzer resulted in data comparable to or better than that achieved when using a vortexer. Additionally, the PortaLyzer works well with various soil types. Our data demonstrate that the troublesome Brown Andisol soils yield higher eDNA concentrations with the PortaLyzer versus the vortexer.

**Table 2:** **DNA Sequencing Quality Comparison**. StErr = Standard Error.

**DNA Extraction Method (Year)**	**# Clusters on Flow Cell Per Barcode**	**Quality Score**	**Number of Raw Reads Generated**	**Number of Unique ASVs After DADA2**
DNEasy PowerSoil Pro Kit with PortaLyzer, plus DNA Clean and Concentrator: full extraction in Iceland (2021)	Average: 543,997 StErr: 35,223	Average: 32.52 StErr: 0.02	10,350,261	6,065,093
DNEasy PowerSoil Pro Kit with PortaLyzer, plus DNA Clean and Concentrator: partial extraction in Iceland (2021)	Average: 633,514 StErr: 47,856	Average: 32.32 StErr: 0.04	12,015,067	6,814,676
DNEasy PowerSoil Pro Kit with vortexer, plus DNA Clean and Concentrator: full extraction in USA (2019)	Average: 90,821 StErr: 6,799	Average: 32.46 StErr: 0.07	1,056,017	868,234

To show that storing samples in Buffer CD2 for one month did not affect bacterial population results, we analyzed each 2021 extraction method separately using the publicly available Qiime2 tool [25]. Below is a breakdown of our Qiime2 analysis pipeline, the script which implements this is available here: https://cluster.earlham.edu/artifacts/static/portalyzer-Iceland-2021-qiime2-run1.sh

In our code, run1 refers to the 2021 extractions that were fully completed in Iceland, while run2 refers to the 2021 extractions that were partially completed in Iceland and stored for 1 month in Buffer CD2 at 4℃. We note that each sequencing run (run1 and run2) were analyzed separately throughout this workflow.

1. Paired end sequence data was obtained from the sequencing core at Indiana University. FWD and REV reads were paired using the *qiime tools import* command, with input-format set to PairedEndFastqManifestPhred33V2.
2. Paired files were run through DADA2 [26] using the command *qiime dada2 denoise-paired*. Primers were removed using the trim function within this command. 89 bp were trimmed from FWD reads and 102 from REV reads. After DADA2 was complete, we retained 57 % of reads from run1 and 61 % of reads from run2.
3. We ran *qiime phylogeny align-to-tree-mafft-fasttree* on each data set to produce the alignments and trees needed for core phylogenetic metrics.
4. We next generated alpha diversity metrics with the command *qiime diversity core-metrics-phylogenetic* with the rooted tree phylogeny produced in step 3 as input. We set the sampling depth for run1 to 209479 (retaining 3,700,622 or 62.17 % of features in 18 samples), and set the sampling depth for run2 to 253244 (retaining 4,558,392 or 61.99 % of features in 18 samples).
5. We ran *qiime feature-classifier classify-sklearn* using the silva-138-99-nb-classifier.qza file available on the Qiime2 website. Barplots of the taxonomy were produced with *qiime taxa barplot*.
6. To examine taxonomy, the assignments were exported into Excel. Read counts for taxonomic units were summed and the top 11 phylum were carried forward for comparison, since these phylum represented more than 95 % of the total classified ASVs.

**Fig. 2:**
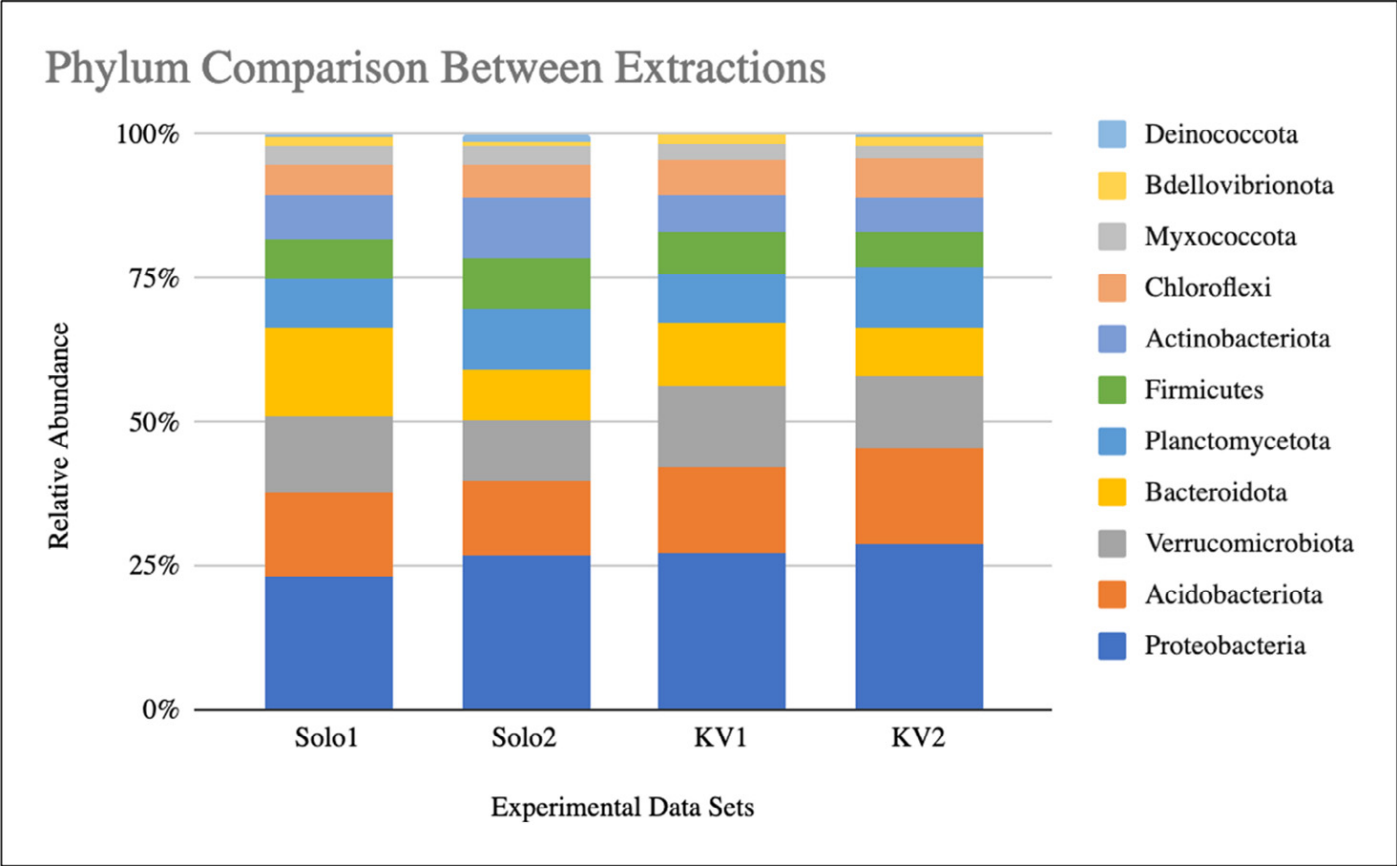
**Comparison of Phylum Across Different Sampling Locations.** The union of the top 10 phylum from each extraction is displayed. Solo1 = First round of DNA extractions from Sólheimajökull (done entirely in Iceland). Solo2 = Second round of DNA extractions from Sólheimajökull (month-long pause). KV1 = First round of DNA extractions from Kvíárjökull (done entirely in Iceland). KV2 = Second round of DNA extractions from Kvíárjökull (month-long pause). Solo1 and KV1 were run on the same flow cell, while Solo2 and KV2 were run on a second flow cell.

We utilized the Qiime2 analysis suite and the SILVA 138 rRNA database to perform taxonomic assignments of our ASVs [25], [27]. We display the top 11 phyla (based on taxonomically classified ASVs) from the four data sets in Figure 2**.** The top 11 phyla represent 95 % of all assigned ASVs. We note the striking similarities between the two rounds of DNA extractions from the glacial forefields. These phyla are expected, as other reports examining eDNA in Iceland and the Arctic find similar compositions [14], [28], [29], [30]. Data for all taxonomy classes is presented in the **supplementary table**.

**Fig. 3:**
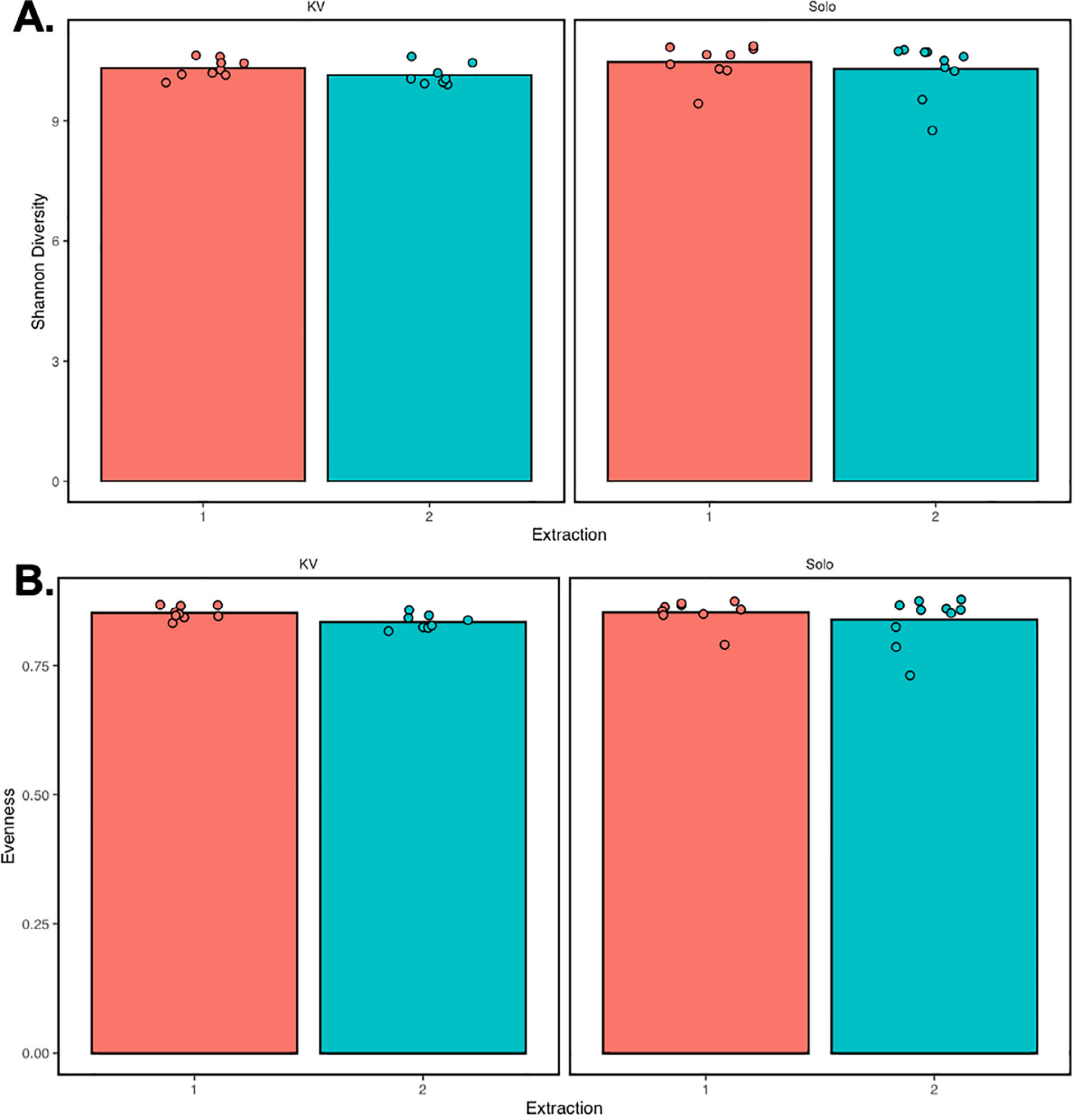
**Alpha diversity metrics from each of the 2021 Iceland data sets.** (A) Shannon Diversity, (B) Pielou’s Evenness. Data from Kvíárjökull (KV) is presented on the left, and data from Sólheimajökull (Solo) is presented on the right. Sequencing runs 1 and 2 are shown in salmon and turquoise, respectively. Solo1 = First round of DNA extractions from Sólheimajökull (done entirely in Iceland). Solo2 = Second round of DNA extractions from Sólheimajökull (month-long pause). KV1 = First round of DNA extractions from Kvíárjökull (done entirely in Iceland). KV2 = Second round of DNA extractions from Kvíárjökull (month-long pause).

To measure how much diversity there is within a particular sample or environment, alpha diversity metrics are employed. Shannon’s Diversity index is a non-phylogenetic metric that combines species richness and their relative abundances [31]. The H-value indicates diversity, with a low H value indicating low diversity and a high H value indicating more diversity. Pielou’s Evenness is a non-phylogenetic metric that measures community evenness, with an output between 0 and 1 [32]. A dominant species results in lower evenness, and thus a lower value. In Figure 3, we show these measurements as calculated for the 2021 samples. These samples were all extracted with the PortaLyzer in place of a vortexer, but the portion of samples in category 1 were extracted without a pause in Buffer CD2, while the samples in category 2 were extracted after being stored in buffer CD2 for one month at 4℃. Shannon’s Diversity and Evenness measurements are almost identical (Figure 3). This indicates that storing samples in buffer CD2 for one month at 4℃ does not change the diversity of communities, aka, it does not create a bias within the experiment.

We have shown that using the PortaLyzer provides DNA extraction with good eDNA yield, leading to high-quality DNA sequencing. Our data indicate that the Portalyzer is a cost-effective, portable, field-ready alternative to a vortexer at the bead-beating step of the DNEasy PowerSoil Pro Kit. Comparisons between soil eDNA extracted with the PortaLyzer and with the commonly used vortexer reveal that the PortaLyzer provides pure DNA capable of generating high-quality sequencing reads (Table 1, Table 2). This indicates that the PortaLyzer can replace a vortexer without loss of DNA concentration or quality. Additionally, we have shown that leaving samples in Buffer CD2 for one month at 4 °C does not negatively impact DNA extraction or sequencing results, in agreement with previous studies [6], [19], [20]. Bioinformatic analysis of the sequenced DNA using Qiime2 shows similarities in taxonomy classifications and alpha diversity between methods (Fig. 2, Fig. 3). We anticipate that longer storage of DNA in Buffer CD2 at 4 °C will be possible without loss of sample quality, since DNA is an extremely stable molecule.

Incorporating the PortaLyzer and cool storage in Buffer CD2 of the DNEasy PowerSoil Pro Kit gives field researchers flexibility and represents additional options for field eDNA extraction protocols. Our field tests have been limited to our work in Iceland and extractions of soil in our local surroundings (southeast Indiana), and we look forward to hearing from researchers who adopt this device and utilize it on other sample types.

**Capabilities and Limitations of Hardware**

• Single battery capacity of approximately 100 minutes using setting 2 with a full load of 2 ml tubes, this yields about 120 samples processed per battery charge. This limitation is easily eliminated with a second battery. We note that we have run the device for over 200 minutes and have not seen any of the parts fail during or after operation.
• Max of 24 samples in 2 mL tubes can be processed at one time.
• Max of 6 samples in 5 mL tubes can be processed at one time.

**Ethics statements**

Our work did not involve animal or human subjects.

## CRediT authorship contribution statement

**Charlie Peck:** Conceptualization, Methodology, Writing – original draft, Writing – review & editing. **Faith Jackobs:** Validation, Writing – original draft, Writing – review & editing. **Emmett Smith:** Conceptualization, Validation, Writing – original draft, Writing – review & editing.

## Declaration of Competing Interest

The authors declare that they have no known competing financial interests or personal relationships that could have appeared to influence the work reported in this paper.
