# Supplementary material for: The PortaLyzer, a DIY tool that allows environmental DNA extraction in the field
Source: HardwareX. 2022 Oct 27;12:e00373. doi: 10.1016/j.ohx.2022.e00373 (PMC9644035; doi:10.1016/j.ohx.2022.e00373)
Supplement: Supplementary data 5 [file mmc5.docx]

**Design files summary**

A three minute video that shows how to assemble the unit from the parts, a one minute video that demonstrates its use in the lab, and a two minute video that demonstrates its use in the field.

| **Design file name** | **File type** | **Open source license** | **Location of the file** |
| --- | --- | --- | --- |
| PortaLyzer Assembly Video | Video | Creative Commons Attribution 4.0 International (CC BY 4.0) | <https://youtu.be/BqmNYpFdPPY> |
| PortaLyzer Lab Usage Video | Video | Creative Commons Attribution 4.0 International (CC BY 4.0) | <https://youtu.be/a0eiRGB9P_o> |
| PoraLyzer Field Usage Video | Video | Creative Commons Attribution 4.0 International (CC BY 4.0) | <https://youtu.be/__26bqYdXGU> |
